# Well-Differentiated Spindle Cell Liposarcoma of the Larynx in a Patient With a History of a Benign Laryngeal Lesion: A Case Report

**DOI:** 10.7759/cureus.93107

**Published:** 2025-09-24

**Authors:** Megan I Watson, Alex L Otto, Randall Hansen, Kent McIntire

**Affiliations:** 1 Otolaryngology - Head and Neck Surgery, A. T. Still University, Kirksville, USA; 2 Internal Medicine - Transitional Year, Hurley Medical Center, Flint, USA; 3 Otolaryngology - Head and Neck Surgery, Freeman Health System, Joplin, USA

**Keywords:** carcinoma of larynx, laryngeal liposarcoma, recurrent laryngeal cancer, spindle cell liposarcoma, well-differentiated liposarcoma

## Abstract

Liposarcomas of the larynx are an extremely rare occurrence. Though they are malignant, their presentation in the larynx may mimic benign conditions; therefore, diagnosis requires histologic evaluation. Our case represents the second known case of laryngeal well-differentiated spindle cell liposarcoma. The patient, a 74-year-old man, presented with dysphagia for two months with a history of a benign laryngeal lesion removed many years prior at another facility. His examination and operative evaluation were initially consistent with a recurrent internal laryngocele, but after pathologic evaluation, the diagnosis of well-differentiated spindle cell liposarcoma was made based on the histologic features as well as cluster of differentiation 34 (CD34) positivity, amplification of murine double minute-2 (MDM2) by fluorescence in situ hybridization (FISH), retained retinoblastoma (RB) protein expression, and negativity for signal transducer and activator of transcription 6 (STAT6). Treatment of these masses typically involves wide local excision, and because they have high rates of recurrence, postoperative surveillance is necessary as well. From this case, it can be concluded that liposarcomas of the larynx should be part of the differential diagnosis of laryngeal masses, even when they appear benign. Due to their rarity, research for risk factors as well as the prognosis of laryngeal liposarcomas is limited, and further studies are needed to better understand these topics and how they affect the diagnosis and treatment of laryngeal liposarcomas.

## Introduction

Liposarcomas are malignant soft tissue tumors that can occur anywhere in the body, though most present in the lower extremities and retroperitoneal regions [1]. Only about 5.6% of liposarcomas occur in the head and neck, with sarcomas of any type representing less than 2% of laryngeal masses [1,2]. In the larynx, liposarcomas are most often found in the supraglottis, with common presenting symptoms including airway obstruction, dysphagia, hoarseness, and throat discomfort [1]. Laryngeal liposarcomas in particular are notorious for appearing benign on exam, which can complicate the initial diagnosis [3]. Definitive diagnosis relies on histopathologic examination of the mass along with the presence of certain immunohistochemical markers to best differentiate between subtypes. Between the four histologic types of liposarcomas, well-differentiated, myxoid/round cell, pleomorphic, and dedifferentiated, the majority of laryngeal liposarcomas are well-differentiated [1]. Spindle cell liposarcoma is a very rare subtype of well-differentiated liposarcomas found in the larynx, characterized by spindle cells containing enlarged nuclei, interspersed with adipocytes, atypical lipogenic cells, and fibromyxoid or collagenous stroma on pathology [4]. Laio et al. (2021) described the only other published case of laryngeal well-differentiated spindle cell liposarcoma (WDSCL), which demonstrated spindle cells intermixed with adipocytes and myxoid features on pathology; however, that case presented in a patient without any significant medical history, unlike the case reported here [5].

Immunohistochemical markers that help determine the final diagnosis include cluster of differentiation 34 (CD34), murine double minute-2 (MDM2), cyclin-dependent kinase 4 (CDK4), and signal transducer and activator of transcription 6 (STAT6). CD34 is a common immunohistochemical marker for soft tissue neoplasms, though it is unreliable for differentiating between benign and malignant lesions, as it may stain positive in both cases [6]. Retinoblastoma protein (RB) expression can be affected in pleomorphic liposarcomas, and may be used to distinguish between types of liposarcomas [7]. Amplification of MDM2 and positivity for CDK4 are more characteristic of well-differentiated liposarcomas in particular, and though a small percentage can be positive for STAT6, that marker is more specific for solitary fibrous tumors (SFTs) or dedifferentiated liposarcomas [4,8]. Because diagnosis of WDSCL largely relies on pathologic evaluation, the recommended treatment involves wide local excision. Distant metastases are uncommon according to the literature; however, considering the high rate of recurrence, these patients should be monitored closely postoperatively [1,3,5]. The goal of this study is to increase awareness surrounding malignancies of the larynx, specifically that they may appear benign, in order to achieve earlier recognition, diagnosis, and treatment.

## Case presentation

**Figure 1:**
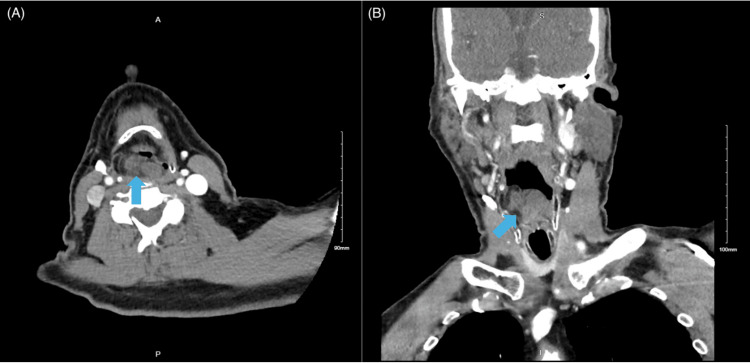
Preoperative axial (A) and coronal (B) CT soft tissue of the neck with contrast showing an 1.8x2.8x2.0 cm right-sided supraglottic soft tissue mass (blue arrows) partially obstructing the larynx.

A 74 year-old man presented to the otolaryngology clinic for dysphagia and jaw pain for the past two months. He was a former smoker, 46.25 pack years, and had a remote history of a presumably benign laryngeal mass that had been removed prior to presentation. Records could not be obtained as the mass reportedly occurred in 1978. A recent CT scan (Figure 1) showed a right supraglottic soft tissue mass that appeared to be consistent with a benign recurrent internal laryngocele, saccular cyst, or fatty deposit. Nasal endoscopy was performed in the office and the mass was visualized on the right supraglottis with a cystic but overall benign-looking appearance. The lesion obstructed much of the right vocal cord, but did not impact vocal cord motion or position. The remainder of his exam, medical history, and review of systems was unremarkable.

Based on the reassuring exam findings and imaging studies, the determination was made that this was likely a recurrence of his prior benign lesion. Treatment options and risks were discussed with the patient and he consented to a wide local excision of the mass in the operating room under direct microlaryngoscopy using a carbon dioxide laser. 

**Figure 2:**
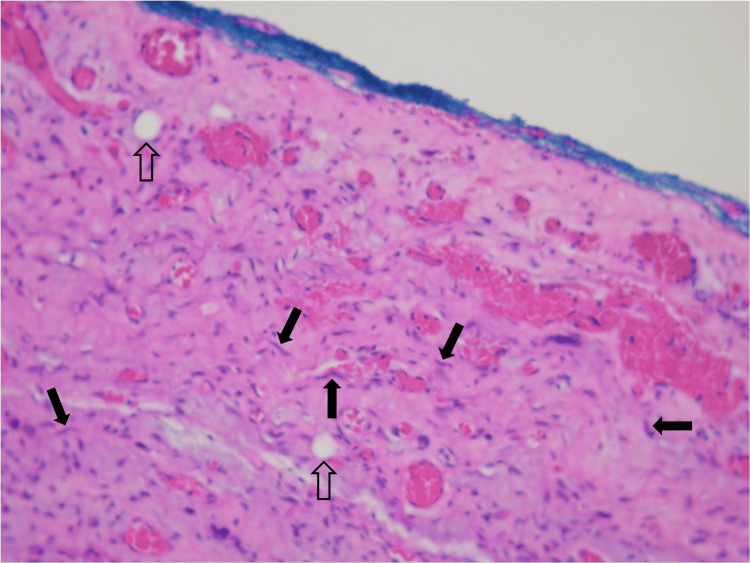
Histopathologic slide of the mass showing spindle cells (solid black arrows) and adipocytes (outlined black arrows) intermixed within a myxoid structure under a hematoxylin and eosin (H&E) stain.

**Figure 3:**
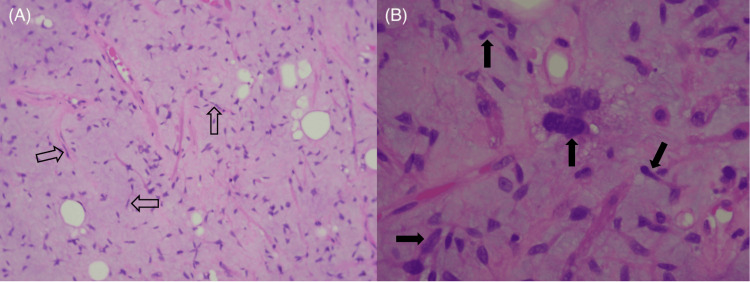
Histologic features of the mass, including views of predominantly spindle cells (outlined black arrows) within the fibromyxoid structure (A) and enlarged, atypical nuclei (solid black arrows) of the spindle cells (B).

The mass was removed from the supraglottis without much difficulty, consistent with the size and behavior of a laryngocele filled with thick, inspissated contents. The initial pathology report for the specimen, however, demonstrated near-complete proliferation of spindle cells within some myxoid fibrous stroma. There were no mitotic figures or necrosis appreciated, but the specimen was sent to a tertiary institution due to its unique pathologic appearance. Upon further review, the mass stained positive for CD34, amplification of the MDM2 gene by fluorescence in situ hybridization (FISH), retained RB expression, and was negative for STAT6 expression. CDK4 expression was not specifically tested; however, these results, in conjunction with the cellular components of the tissue, resulted in a diagnosis of well-differentiated spindle cell liposarcoma. Histologic images of the mass are shown in Figures 2, 3.

While the mass was grossly removed in its entirety in the operating room and sent as a permanent specimen due to its benign appearance, the pathologic evaluation later returned demonstrating an extensive positive margin. The patient was presented at a multidisciplinary sarcoma tumor board as a result, which recommended referral to a tertiary facility with an immediate consult to radiation oncology for further treatment. One month after surgery, the patient had a positron emission tomography (PET) scan that was unremarkable. The patient was monitored every three months postoperatively, but developed a recurrence five months later that encompassed the right arytenoid and extended along the right aryoepiglottic fold. Noted on the subsequent flexible nasolaryngoscopic exam was possible post-cricoid involvement versus edematous mucosa. For treatment of the recurrence, the patient underwent a total laryngectomy with adjuvant radiation at a tertiary facility, nine months after the initial resection. The pathology report from the second procedure demonstrated well-differentiated liposarcoma with extensive myxoid features. More detailed records could not be obtained to determine whether or not the recurrent specimen had a similar, distinct spindle-cell morphology to the presenting lesion.

## Discussion

Laryngeal liposarcomas can appear similarly to many benign conditions of the larynx, including but not limited to lipomas, laryngoceles, fibromas or fibromatosis, myofibroblastic tumors, and other types of sarcomas, such as myxofibrosarcomas and fibromyxoid sarcomas [3,4]. They may resemble these other conditions in shape, size, color, consistency, location, or growth pattern, to name a few. The multitude of conditions that laryngeal liposarcoma can mimic makes diagnosis challenging with physical exam alone, possibly delaying treatment and resulting in a reliance on pathologic evaluation in order to make an accurate diagnosis. Along with the histologic appearance of the mass, this evaluation should include testing for expression of CD34, CDK4, MDM2, STAT6, and RB to distinguish well-differentiated liposarcomas from other conditions, such as laryngeal lipomas, solitary fibrous tumors, dedifferentiated liposarcomas, and pleomorphic liposarcomas [4,6-8]. The WDSCL subtype shows significant proliferation of atypical spindle cells on histology and stains positive for CD34 and MDM2 expression [4,6]. Well-differentiated liposarcomas should also stain negative for STAT6 and have no loss of RB expression, unlike the dedifferentiated and pleomorphic liposarcoma subtypes, respectively [7,8]. CDK4 was not specifically tested in this case, but because MDM2 and CDK4 are closely related, tumors that are positive for MDM2 amplification are often also positive for CDK4. Therefore, in the setting of MDM2 amplification and with the other markers described above, a diagnosis of WDSCL could still be made without CDK4 testing [4]. Imaging studies can help determine the extent of these lesions for preoperative planning, but do not offer much insight for diagnosis.

Treatment of laryngeal liposarcomas relies primarily on complete, wide local excision with negative margins [5]. While laryngeal liposarcomas are known to have high recurrence rates, regional lymph node spread and distant metastasis are rare, and there is limited evidence supporting adjuvant radiation and/or chemotherapy when complete excision has been achieved [1,4]. Prognosis is not well-studied in patients with laryngeal liposarcoma and guidelines for postoperative surveillance are not well established due to its rarity. Smoking may be a risk factor for this condition, as it is a known risk factor for head and neck cancer in general and present in the case presented [9]. However, more studies would be necessary to determine the relationship between smoking and the incidence of laryngeal liposarcoma, or to investigate other risk factors for this condition.

One limitation of this report is the inability to obtain records or gather more history from the patient's reported benign lesion. The remote history supports the patient's report that the mass was benign, but a malignancy cannot be completely ruled out in the absence of the records. Along a similar vein, not having access to the pathology records from the recurrence resulting in this patient's total laryngectomy means we cannot confirm that this was a true recurrence of his same mass as opposed to a de novo malignancy. A final limitation is the lack of testing for CDK4. While the immunohistochemical markers present were satisfactory to make a diagnosis of WDSCL in our case, a test for CDK4 could have further supported this diagnosis and may help practitioners recognize this condition in the future if their pathology demonstrates a positive CDK4.

The case presented above adds to the limited literature discussing WDSCL of the larynx and highlights the importance of maintaining a broad differential diagnosis for laryngeal masses. Given the rarity of laryngeal WDSCL, as well as its ability to mimic benign lesions, patients presenting with nonspecific complaints and benign-appearing laryngeal masses such as this case should undergo thorough evaluation for proper diagnosis. We, as the authors, intend that with increased awareness of laryngeal WDSCL and contributions to the literature, recognition and early diagnosis will improve, postoperative care may be standardized, and the patients with this condition will receive higher quality care as a result.

## Conclusions

Laryngeal liposarcoma is a rare condition, with well-differentiated spindle cell liposarcoma of the larynx only described in the literature in one other case. Thorough pathologic evaluation is required for a definitive diagnosis of spindle cell liposarcoma, including the characteristic CD34 and MDM2 expression. Treatment primarily consists of wide local excision of the lesion with negative margins, and because of the high risk of recurrence, patients should be monitored closely in the postoperative period. More studies are necessary to evaluate risk factors and prognosis of patients with this condition.
